# Two novel mutations in *PADI6* and *TLE6* genes cause female infertility due to arrest in embryonic development

**DOI:** 10.1007/s10815-021-02194-1

**Published:** 2021-05-26

**Authors:** Juan Liu, Zongjian Tan, Jun He, Tingting Jin, Yuanyuan Han, Li Hu, Shengwen Huang

**Affiliations:** 1 School of Medicine, Guizhou University, Guiyang, China; 2 Prenatal Diagnosis Center, Guizhou Provincial People’s Hospital, Guiyang, China; 3 Department of Reproductive Medicine, Guizhou Provincial People’s Hospital, Guiyang, China; 4 NHC Key Laboratory of Pulmonary Immunological Diseases, Guizhou Provincial People’s Hospital, Guiyang, China

**Keywords:** Female infertility, Embryonic developmental arrest, Subcortical maternal complex, Mutation

## Abstract

**Purpose:**

This study aims to identify genetic causes of female infertility associated with recurrent failure of assisted reproductive technology (ART) characterized by embryonic developmental arrest.

**Methods:**

We recruited infertile patients from two consanguineous families from the Reproductive Medicine Center of Guizhou Provincial People’s Hospital. Peripheral blood was collected for genomic DNA extraction. Two affected individuals and their family members were performed with whole-exome sequencing and Sanger validation in order to identify possible causative genes. For further analyzing the effect of splicing mutation on mRNA integrity in vivo, *TLE6* cDNA from the peripheral blood lymphocyte of the affected individual was sequenced. In addition, the possible impact of the pathogenic mutation on the structure and function of the protein were also assessed.

**Results:**

Two novel homozygous mutations in the peptidylarginine deiminase type VI (*PADI6*) and the transducin-like enhancer of split 6 (*TLE6*) genes were identified in the two families. One patient carried the frameshift deletion mutation c.831_832del:p.S278Pfs^*^59 of the *PADI6* gene and the other patient carried the splicing mutation c.1245-2 A>G of the *TLE6* gene. The analysis of the mRNA from the proband’s peripheral blood leukocytes confirmed aberrant splicing.

**Conclusions:**

Our findings expand the mutational spectrum of *PADI6* and *TLE6* associated with embryonic developmental arrest and deepen our understanding of the genetic causes of infertility with recurrent ART failure.

## Introduction

Assisted reproductive technology (ART), mainly including in vitro fertilization (IVF) and intracytoplasmic sperm injection (ICSI), is an important milestone in the development of medicine and life science. The increasing number of infertile couples has caused the demand for the development of ART. It has been estimated that the number of babies delivered worldwide as a result of IVF and other advanced fertility treatments is more than 8 million. However, part of the patients who have attempted reproduction via ART multiple times have failed for unknown reasons. Normal gamete formation, fertilization, and embryonic development are fundamental factors in successful human reproduction. Abnormalities in any part of the process lead to IVF/ICSI failure. Recent studies have demonstrated that several mutant genes are associated with infertility, such as *PATL2* (OMIM: 614661) [1–4] responsible for oocyte maturation arrest, *WEE2* [5] leading to fertilization failure, *ZP3* [6], which causes recurrent empty follicle syndrome, and *TUBB8* [7–10], which results in variant phenotypes covering oocyte maturation, fertilization, and zygote cleavage. Recently, mutations in *PADI6* and *TLE6* which encode the proteins involved in the subcortical maternal complex (SCMC) were shown to be responsible for human early embryonic lethality [11, 12]. Nevertheless, until now, the genetic determinants that cause early human embryonic arrest have remained elusive.

Following fertilization, via maternal-to-zygotic transition (MZT), the zygotic genome is activated and embryogenesis is mobilized [13]. Accurate embryonic development is a crucial step contributing to a successful pregnancy. It is well known that maternal effect factors play a critical role in the early stage of embryonic development. The SCMC, which is encoded by maternal effect genes (MEGs), is a multiprotein complex exclusively expressed in mammalian oocytes and early embryos, essential for embryonic cell divisions [14]. The SCMC is composed of nine proteins as follows: transducin-like enhancer of split 6 (TLE6); peptidylarginine deiminase, type VI (PADI6); NLR family pyrin domain-containing 5 (NLRP5); NLR family pyrin domain-containing 2 (NLRP2); NLR family pyrin domain-containing 7 (NLRP7); oocyte expressed protein (OOEP); KH domain-containing protein 3 (KHDC3L); zinc finger, BED-type containing 3 (ZBED3); and NLR family, pyrin domain containing 4F(Nlrp4f) which are encoded by *TLE6*, *PADI6*, *NLRP5*, *NLRP2*, *NLRP7*, *OOEP*, *KHDC3L*, *ZBED3*, and *Nlrp4f*, respectively [15–18]. Previous studies indicated that the SCMC via cofilin controlled the formation of the actin cytoskeleton which regulated the central position of the spindle and caused certain symmetric division of mouse zygotes [19]. Therefore, anomaly of SCMC would result in abnormal cell division and possibly embryonic arrest. So far, there have been just a few reports on human embryonic arrest caused by SCMC abnormity associated with the above nine gene mutations [12, 20–23].

In the current study, two novel homozygous mutations were identified in *PADI6* and *TLE6* by whole-exome sequencing (WES) as a cause of recurrent ART failure in infertile patents from two consanguineous families. The findings suggest the potential involvement of SCMC in embryo development and extend the mutational spectrum of SCMC.

## Materials and methods

### Study subjects

Two infertile patients with recurrent failure of ART attempts from two consanguineous families were recruited from the Reproductive Medical Center of the Guizhou Provincial People’s Hospital. The present study was approved by the Ethics Committee of the Guizhou Provincial People’s Hospital. Written informed consent was obtained from all participants.

### WES and variant analysis

Genomic DNA samples of affected individuals and their family members were extracted from peripheral blood leukocytes using the Blood Genomic DNA kit (Tiangen, Beijing, China). WES was carried out on the probands and other family members. The Illumina sequencing library was constructed following sample preparation and in the next step, the biotin-labeled whole exome probes hybridized the DNA for capturing and enriching the desired target genes. Subsequently, next-generation sequencing was performed using the Illumina Nextseq 500 sequencer (Illumina, San Diego, USA). Sequencing reads were aligned to the human genome GRCh37/hg19 by using Burrows–Wheeler Alignment (BWA). Single-nucleotide variants (SNVs) and small indels were identified using the SAM tool (http://samtools.sourceforge.net/). Following removal of the redundant reads during PCR amplification, SNVs and indels in each sample were recalibrated and named with the GATK program. The identified variants were annotated and classified with the ANNOVAR software. The variants were prioritized based on the following filtering criteria: (1) The variations with minor allele frequencies <0.1% were identified from the following databases: exome aggregation consortium (ExAC), 1000 Genomes Project, and Genome Aggregation Database (Genome AD). (2) Synonymous and non-coding variants not predicted to affect splicing were excluded. (3) Variants with an embryogenesis-related function. (4) Location in or near a homozygous region higher than 2.0 Mb. Meanwhile, the pathogenicity of each genetic variation was classified based on the Online Mendelian Inheritance in Man (OMIM) (https://www.omim.org/), Human Gene Mutation Database (HGMD) (http://www.hgmd.cf.ac.uk/ac/index.php), ClinVar database (https://www.ncbi.nlm.nih.gov/clinvar/), and the American College of Medical Genetics and Genomics (ACMG) and the Association for Molecular Pathology (AMP) guidelines.

### Sanger sequencing

Specific primers flanking the mutations in the *PAID6* and *TLE6* genes were designed by Primer 5.0 and used for PCR amplification with an ABI 3130 Genetic Analyzer (Foster City, California, USA). Subsequently, sequence analysis was performed using the Chromas program in the DNASTAR analysis package. The variants of the target genes were verified by Sanger sequencing in all of the individuals.

### Evolutionary conservation and in silico analysis

Conservation analysis of the mutated amino acid sequences was performed using the Clustal Omega (https://www.ebi.ac.uk/Tools/msa/clustalo/). The three-dimensional structures and structural diversity analysis of WT and mutant (PADI6) proteins were predicted using the Phyre2 software (http://www.sbg.bio.ic.ac.uk/phyre2/html/page.cgi?id=index) and conducted with Swiss-PdbViewer 4.04.

## RT-PCR and cDNA sequencing

Total RNA was isolated from the peripheral blood leukocytes of the proband in family 2 and from one normal control by the Trizol Reagent (Thermo Fisher Scientific, Inc.). The RNA was reverse-transcribed using reverse transcriptase (TaKaRa PrimeScript reagent kit) and amplified by PCR with Taq polymerase. The primers spanned exons 13–15 of *TLE6* exons and had the following sequences: *TLE6* forward, 5′ CACCTGCCTGCTGTCCTCAAA 3′; and *TLE6* reverse, 5′ GGCGGTTGTTGGAAGAGACGT 3′. The PCR products were detected by electrophoresis and further cloned into the TA cloning vector. Individual clones were isolated and sequenced using ABI 3130 Genetic Analyzer (Foster City, California, USA).

## Results

### Clinical characteristics of the patients

We identified two novel mutations in infertile patients from two disparate consanguineous families with recurrent failure of ART attempts. The affected individuals had been diagnosed with primary infertility for several years and had experienced multiple failed IVF/ICSI cycles in different hospitals. Their spouses demonstrated normal sperm counts, sperm morphology, motility, and acrosin activity.

**Fig. 1:**
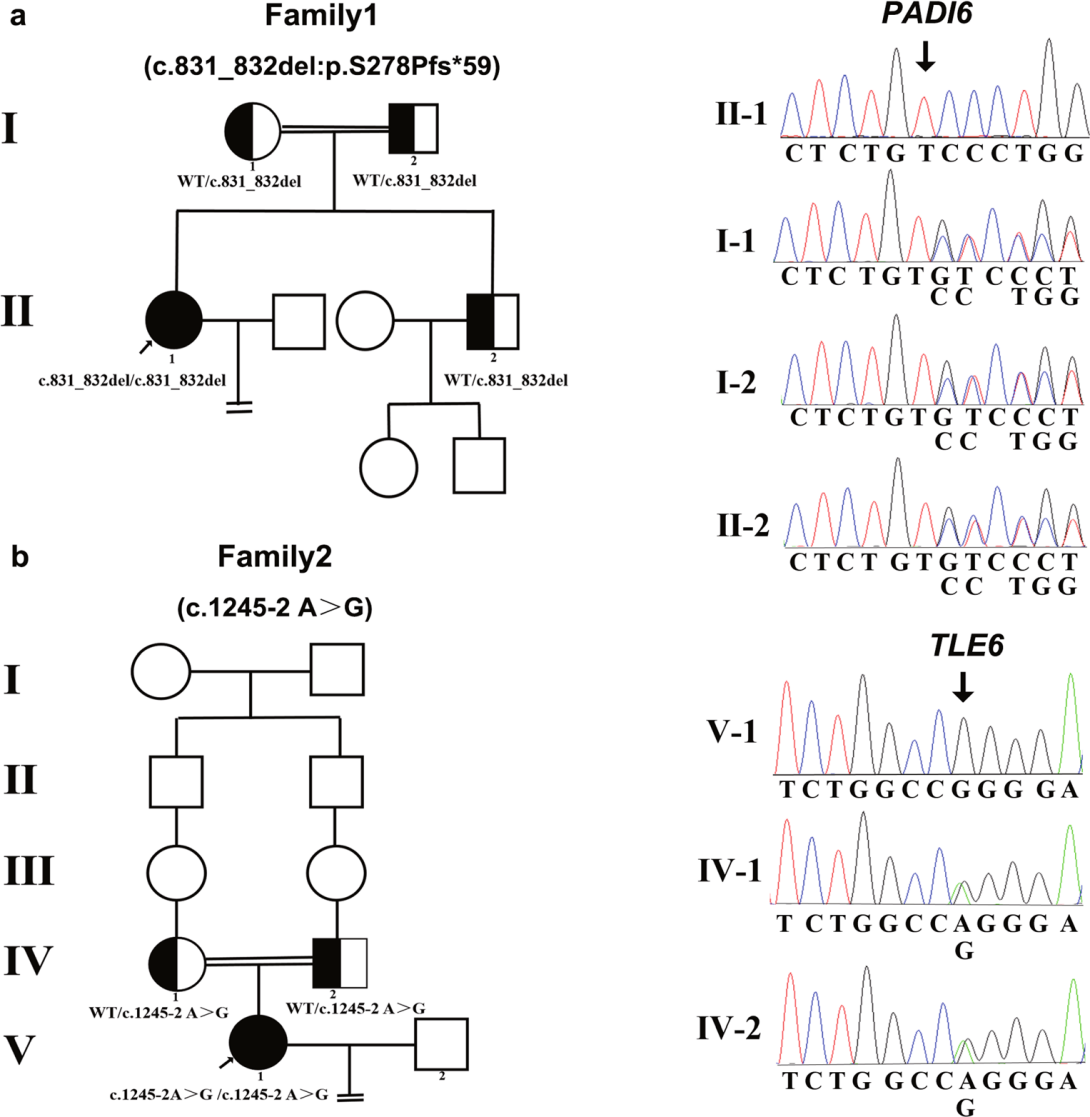
Identification of *PADI6* and *TLE6* mutations in two affected families. **a** The pedigree of family 1 is affected by the mutation of *PADI6.* **b** The pedigree of family 2 carrying the *TLE6* mutation. The two affected individuals exhibited homozygous mutations with a recessive inheritance pattern. Sanger sequencing confirmation was demonstrated next to the pedigrees. Black circles with arrows indicate the affected individuals. Semi-filled symbols indicate the heterozygous mutation carriers. The double lines denote consanguinity and the equal signs represent infertility

**Fig. 2:**
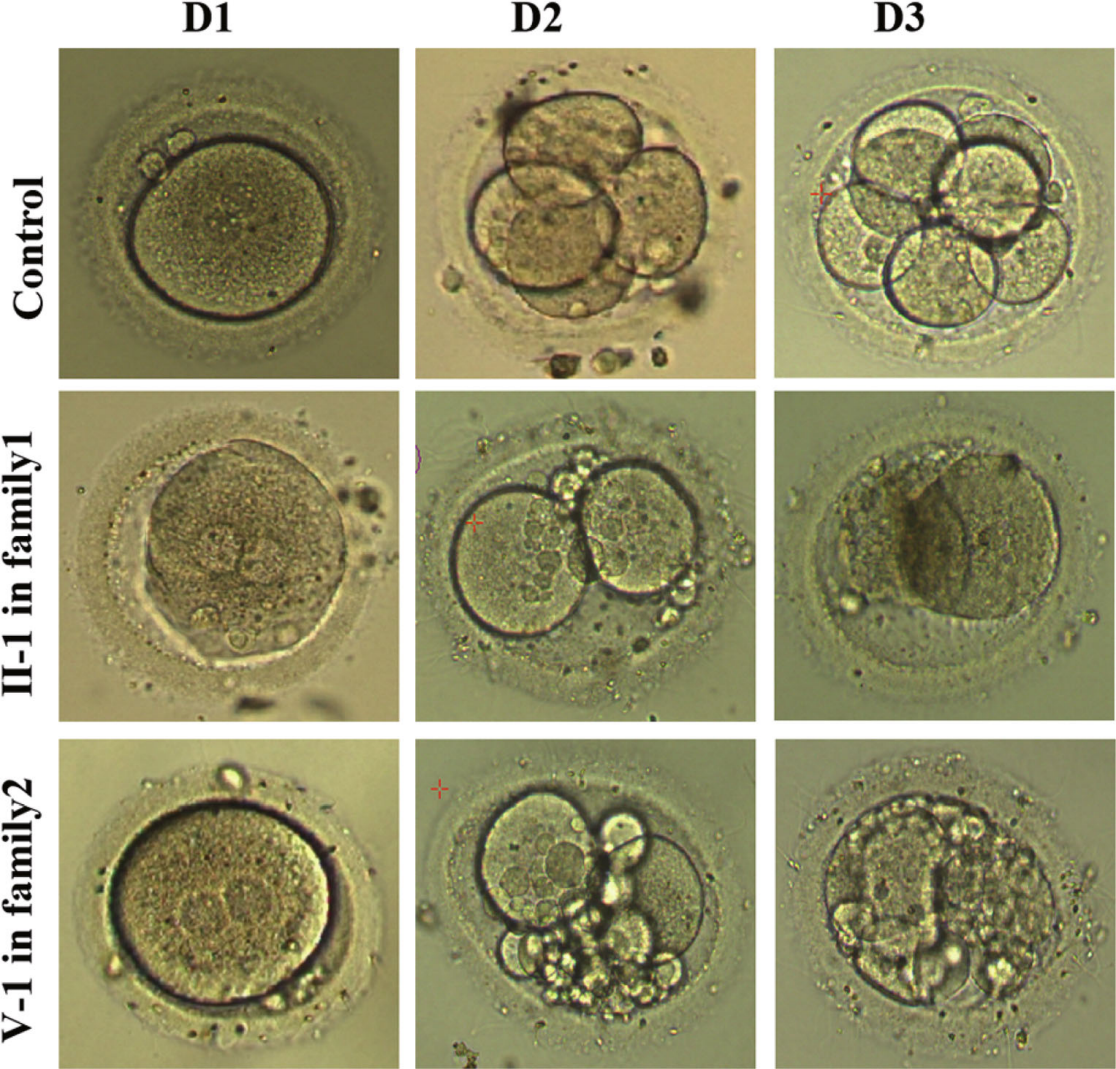
Phenotype of oocytes/embryos from the patients from two families. The morphology of the oocytes and embryos derived from a control , the patient II-1 in family 1, and patient V-1 in family 2, respectively

The proband of the family 1 was 28 years old with a 2-year history of primary unexplained infertility. She was from a consanguineous family (Fig. 1a) and had undergone two trials of failed IVF and ICSI cycles. Initially, the couple underwent three cycles of letrozole combined with intrauterine insemination. However, the subjects failed to become pregnant. In light of the unexplained infertility, ART treatment was provided. In the first attempt, a gonadotropin-releasing hormone antagonist (GnRHa) protocol was performed. Ten oocytes were retrieved and six of these were fertilized, but only three were processed normal fertilization (2PN). However, no viable embryos were obtained on day 3 and all of them were arrested at the two- to three-cell stage (Fig. 2). In the second ICSI attempt, a long protocol was utilized and 10 MII out of 14 oocytes were obtained, of which 6 oocytes were fertilized. However, all the zygotes developed into poor-quality embryos on day 3 and one 5-cell embryo was transferred but failed to induce pregnancy (Table 1). Other embryos failed to develop into blastocysts following prolonged cultivation (Table 1).

**Table 1:** Oocyte and embryo characteristics of IVF and ICSI attempts of the two patients

Patient	Age	Duration of infertility years	IVF/ICSI attempts	COH protocol	Oocytes retrieved	Fertilized oocytes	Embryos transferred	Outcomes
II-1 in family 1	28	2	1st IVF	GnRHa	10	6 (3 2PN/2 1PN/1 3PN)	0	All the embryos arrested on day 3
			2nd ICSI	Long	14	6 (6 2PN)	1	The 2PN zygotes were cleaved to 1 grade II 5-cell stage for transfer but failed to establish pregnancy, 2 grade II 4-cell stage, and the remaining 3 embryos showed arrested on day 3. All embryos arrested during subsequent blastocyst culture
V-1 in family 2	32	7	1st IVF	GnRHa	6	5 (4 2PN/1 1PN)	1	One embryo viable was transferred but failed to establish pregnancy, the remaining embryos arrested at 2 and 6 cells on day 3
			2nd IVF	GnRHa	5	4 (4 2PN)	0	The 2PN zygotes were cleaved to 2 grade III 4-cell embryos and two 2-cell stage arrested

The proband of family 2 was 32 years old with a 7-year history of primary infertility and had four failed IVF attempts. She was from an another consanguineous family and her parents were second cousins (Fig. 1b). The female individual presented with irregular menstrual cycles and anovulation during her initial visit in our reproductive center. Her failure to conceive was followed by three cycles of ovulation induction with letrozole. Due to the co-existing left fallopian tube obstruction, the couple accepted the IVF treatment. In the first IVF attempt, a GnRHa protocol treatment was used and six oocytes were retrieved, of which five were fertilized successfully. However, no high-quality embryos were available on day 3. Only one viable embryo (a grade II 7-cell embryo) was transferred but failed to establish pregnancy and the remaining embryos were arrested at the 2- to 6-cell stage. In the second IVF attempt, four out of five oocytes were successfully fertilized but no viable embryo was acquired. The embryos grew into poor-quality embryos and all were arrested during further blastocyte cultivation until day 5 (Fig. 2, Table 1). According to the statement offered by the individual, no embryos were viable in the subsequent two IVF cycles performed in other hospitals with different treatment protocols due to the arrest of all the embryos.

### Identification of novel mutations in *PADI6* and *TLE6*

**Fig. 3:**
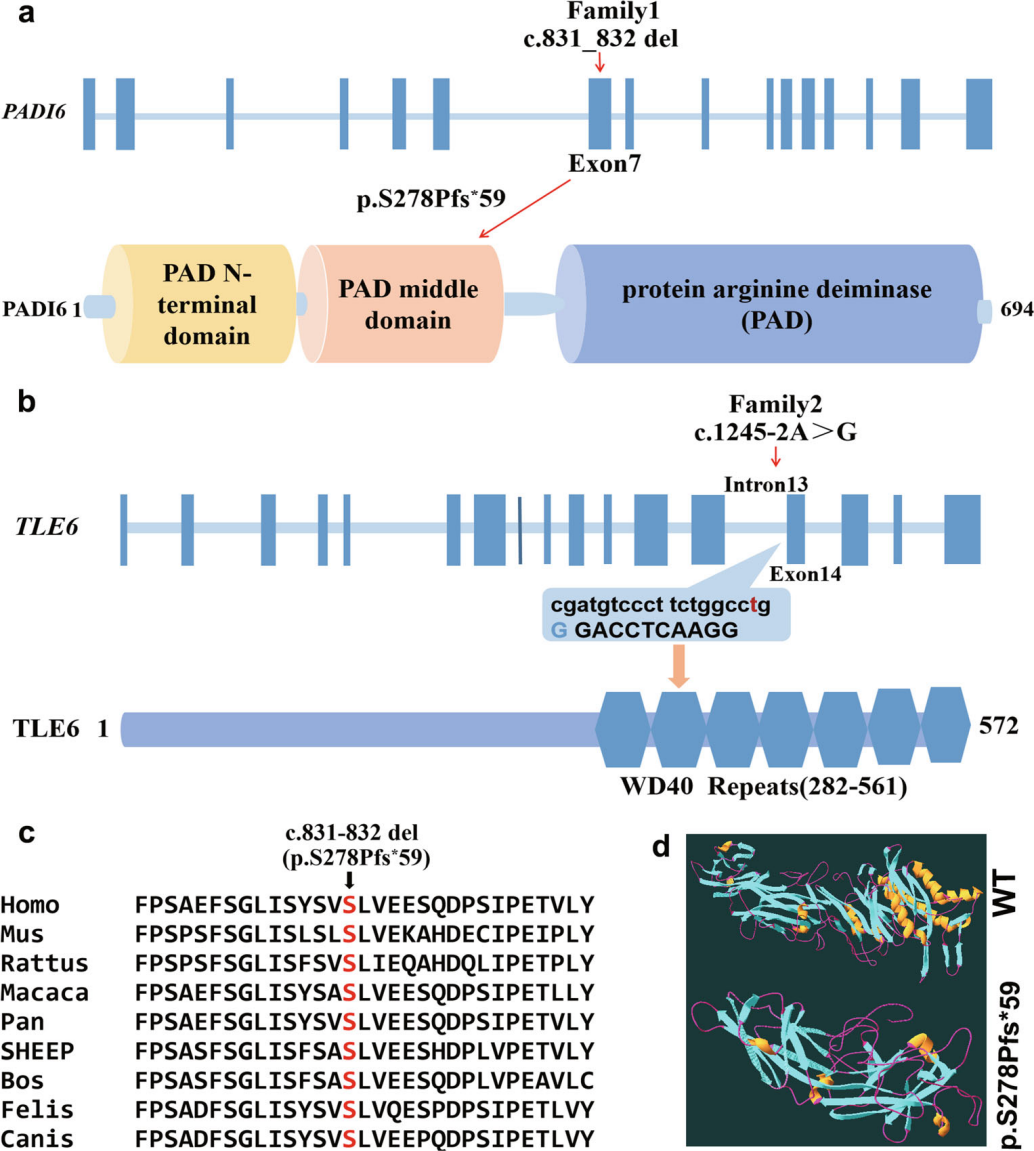
The locations and conservation of mutated residues in *PADI6* and *TLE6*. **a** The position of the mutation is indicated in the genomic structure and protein structure of *PADI6.* **b** Localization of the mutation in the genomic and protein structure of *TLE6.* **c** Conservation of mutated amino acids in PADI6 among 9 different species. The residues S278 are highly conserved across species. **d** Prediction of the effect of mutation in PADI6 on protein conformation. The view shows the structure comparison of the frameshift deletion mutation PADI6 protein with wild-type PADI6 protein

In family 1, a homozygous frameshift deletion mutation was identified in exon 7 of *PADI6* (NM_207421.4; c.831_832del:GT; p.S278Pfs^*^59) in the proband. Her parents and brother carried a heterozygous *PADI6* mutation and the mutation was verified by Sanger sequencing, which confirmed that p.S278Pfs^*^59 was from her parents (Fig. 1a). In family 2, the patient had a homozygous splicing mutation in intron 13 of *TLE6* (NM_001143986.1; c.1245-2 A>G) and her parents had a heterozygous *TLE6* mutation, as determined by Sanger sequencing (Fig. 1b). The location of *TLE6* is indicated in Fig. 3b. Both of the mutations indicated a recessive inheritance pattern. Moreover, these two mutations were not found in the East Asian population of the ExAC, 1000 Genomes, and GnomAD database. According to the ACMG/AMP standards and guidelines, the classification of the frameshift deletion variant in family 1 was pathogenic and the criteria included PVS1, PM2, and PP4. The pathogenic variant was connected with preimplantation embryonic lethality 2, which is an autosomal recessive hereditary disease. Similarly, the classification of the splicing variant in family 2 was considered pathogenic by the ACMG/AMP standards and guidelines and the criteria included PVS1, PM2, and PP4. The splicing mutation was associated with preimplantation embryonic lethality known as an autosomal recessive hereditary disease.

## Results of conservative and in silico analysis

The amino acids at position p.S278 of PADI6 were highly conserved in nine species, suggesting that the mutation was likely pathological. The location of the *PADI6* mutation and the conservation analysis among different species are shown in Fig. 3a and c. According to a three-dimensional (3D) structure of PADI6, the frameshift deletion mutation caused a replacement of serine with proline at position 278, further leading to the production of truncated protein compared with the WT type (Fig. 3d).

## *TLE6* RNA analysis of peripheral blood of proband in family 2

**Fig. 4:**
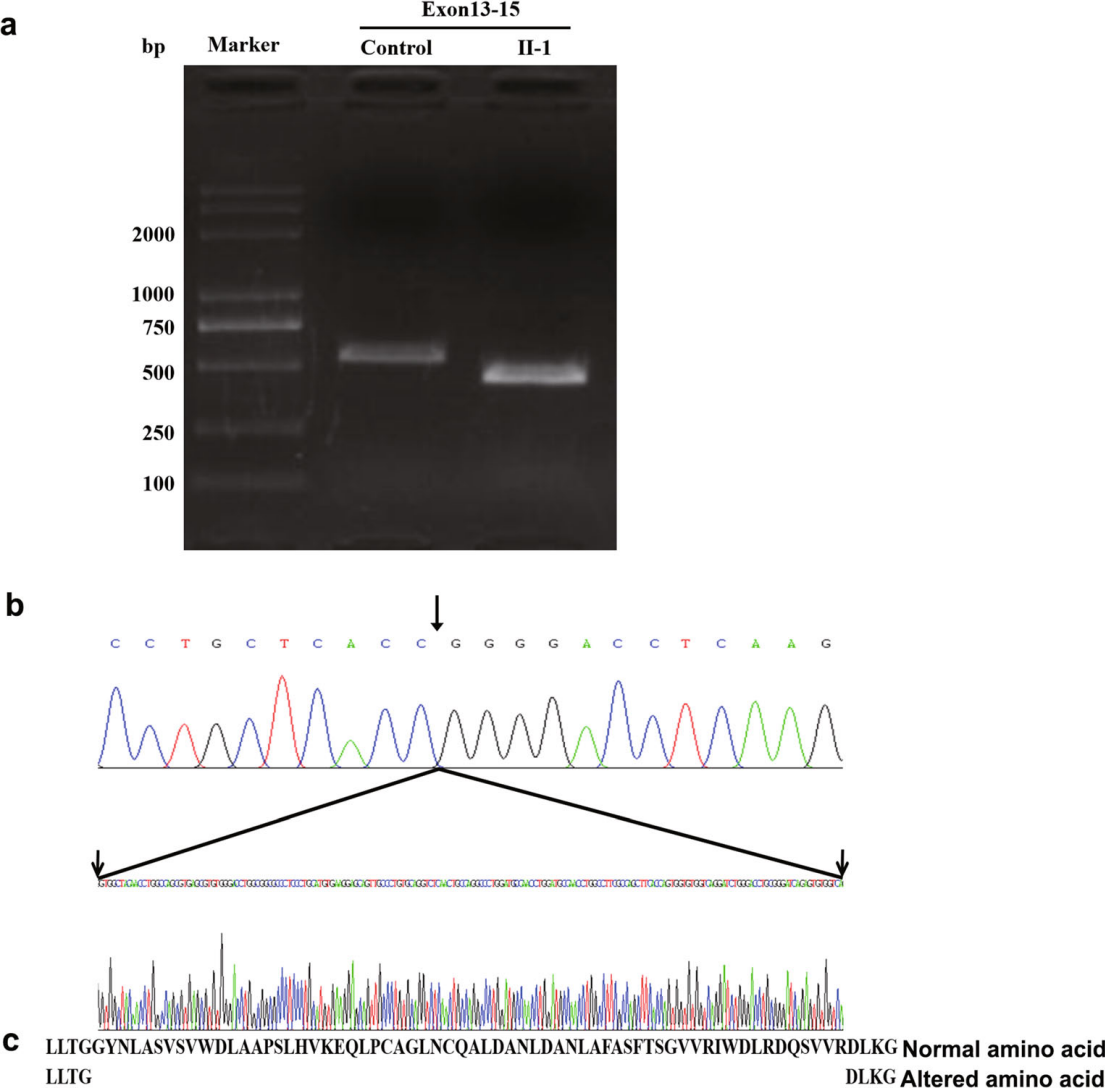
Sequence chromatograms of cDNA of the *TLE6* gene. **a** Electrophoresis of cDNA amplified from the affected individual in family 2 and cDNA from a normal individual (control). The samples of the patient indicated a large deletion fragment compared with the control subject. **b** The top and lower sequence chromatograms represent the cDNA sequence of the proband and from a normal control, respectively. The sequence between the two arrows indicates the 189-bp deletion of the patient compared with the normal control. **c** The amino acid sequence of normal and mutant TLE6 proteins

To explore the effects of splicing mutations on mRNA integrity in vivo, we extracted RNA from the blood of the proband in family 2 and one normal control and amplified the mutation region by reverse transcription-polymerase chain reaction (RT-PCR). The results indicated that a truncated amplification band appeared in the patient’s PCR product compared with that of the control (Fig. 4a). Subsequently, we sequenced *TLE6* cDNA by using TA-clone libraries and found that the truncated product lacked a 189-bp sequence (Fig. 4b). Aberrant splicing resulted in the loss of the majority of the bases of exon 13 and the deletion of 63 amino acids in the corresponding code (Fig. 4c). These changes were confirmed by sequence alignment and were not found in the control individual.

## Discussion

In the present study, two novel homozygous mutations in the SCMC genes *PADI6* and *TLE6* were identified from two independent patients with recurrent ART failure. The majority of the embryos indicated developmental arrest and part of the zygote developed into poor-quality embryos that failed to establish pregnancy.

*PADI6* is a crucial member of SCMC, which has been reported to result in female sterility and is associated with a phenotype of early human embryonic arrest possibly due to impairment of embryonic genome activation (ZGA) [24]. The transition from maternal to embryonic control and transcriptional activation of the newly formed embryonic genome is characterized by ZGA and plays a crucial role in the process of normal embryonic development. An increasing number of functional studies in mice have reported that oocytes from *Padi6*^−/−^ lack a structure unique to mammalian oocytes and preimplantation embryos named cytoplasmic lattices (CPLs) in which ribosomal components are stored have abnormal organelle positioning and redistribution, reduced de novo protein synthesis, and eventually result in early arrested embryonic development [15, 25, 26]. *PADI6* comprises 16 exons which encode a protein containing 694 amino acids. The peptidylarginine deiminase (PADI) family is composed of five members (PADI1–PADI4 and PADI6) and PADI6 is exclusively expressed in oocytes and early embryos [27]. The majority of the mutations identified in previous studies were located in the PAD domain and only one compound-heterozygous mutations (p.H211Q and p.E670Gfs*48) was noted in the PAD middle domain [21, 23, 24, 28, 29]. In the present study, a homozygous mutation was identified, which was located in the PAD middle domain, leading to arrest in embryonic development. Previous studies have shown that mutations in *PADI6* can cause different phenotypes with regard to embryo developmental arrest between the 2- and 5-cell stage. In some cases, embryos failed to form blastocysts and even a small amount of viable embryos were produced on day 3, or the embryos were transferred into the uterus but led to pregnancy failure [21, 23, 24]. In addition, a recent study demonstrated that the missense variants in *PADI6* caused comparative mild phenotypes including miscarriages and molar pregnancies. However, the pregnancy only lasted up to 8–12 weeks [29]. In the present study, the novel homozygous frameshift mutation c.831_832del (p.S278Pfs*59) was identified in *PADI6.* The *PADI6* variants result in a premature stop codon, leading to a truncated protein. The patients in our study demonstrated embryo arrest between the zygote and the 6-cell stage on day 3, whereas embryos failed to form blastocysts on day 6 and a low-quality embryo was transferred into the uterus, but failed to establish a pregnancy which was consistent with the results reported previously. As far as we know, this is the first report investigating the homozygous mutation in the PAD middle domain.

*TLE6* is another maternal effect gene that encodes a member of the SCMC in mammalian oocytes. In an animal model, it has been demonstrated that the deficiency of Tle6 impacts the dynamics of F-actin via cofilin in the mouse zygote and results in asymmetric cell division and cleavage-stage embryonic lethality [19]. Human TLE6 possesses 44% identity with the mouse homolog and it was primarily identified primarily in the subcortex of oocytes and early embryos [20]. In 2015, a single mutation in *TLE6* was recognized as causing the earliest known human embryonic lethality via reducing the potential effect for PKA-catalyzed phosphorylation and inhibiting its binding to SCMC components [12]. In the present study, a novel homozygous splicing mutation in *TLE6* was identified from a patient with recurrent ART failures. The phenotype of the patient indicated that the oocyte could be fertilized normally but subsequently the majority of embryos were arrested on day 3 and only one low-quality embryo was used for transfer in multiple treatment cycles, which exhibited a similar phenotype with that of the maternal mouse *Tle6* knockout. Three previous studies identified six different mutations in *TLE6*, comprising three homozygous missense mutations (c.1529C>A:p.S510Y;c.1226G>A:p.Arg409Gln;c.1621G>A:p.Glu541Lys), one homozygous frameshift mutation (c.1133del: p.A378Efs*75), and two compound heterozygous missense mutations (c.388G>A:p.Asp130Asn and c.1507G>A:p.Val503Ile) [12, 21, 22]. At present, one additional novel *TLE6* mutation was identified, which further expanded the mutation spectrum of *TLE6*. In the present study, the mutation occurred at the canonical acceptor splicing site, which was localized at intron13 terminal upstream 2 base pair (c.1245-2 A>G). Following subsequent verification of the mRNA alteration, the data demonstrated a 189-bp deletion in exon 13. Consequently, the splicing mutation triggered exon skipping and resulted in protein truncation.

In conclusion, the present study identified novel mutations in the SCMC genes *PADI6* and *TLE6* and expanded the spectrum of genetic causes of female infertility with recurrent ART failure characterized by embryonic arrest in development.

## Declaration

The study was approved by the Ethics Committee of the Guizhou Provincial People’s Hospital (No. 21/2019).
